# Occipital Nerve Blockade for the Treatment of Occipital Neuralgia-Like Acute Postcraniotomy Headache: A Retrospective Study

**DOI:** 10.1155/2021/5572121

**Published:** 2021-04-19

**Authors:** Shaoheng Wang, Xueye Han, Chunmei Zhao, Fang Luo

**Affiliations:** ^1^Department of Anesthesiology, Beijing Shijitan Hospital, Capital Medical University, Beijing 100038, China; ^2^Department of Pain Management, Beijing Tiantan Hospital, Capital Medical University, Beijing 100070, China

## Abstract

**Objective:**

The therapeutic effectiveness and safety of occipital nerve blockade (ONB) on occipital neuralgia- (ON-) like acute postcraniotomy headache (ON-APCH) was evaluated.

**Background:**

Persistent occipital neuralgia is a subclassification of chronic postcraniotomy headache and has been investigated sporadically in previous publications. The long-lasting neuralgic pain significantly impairs postoperative recovery and quality of life. However, little is known regarding ON-APCH and its management.

**Methods:**

All data were retrospectively acquired from consultation records and electronic institutional medical documents. Forty-one patients, who developed drug-resistant ON-APCH after elective craniotomy and received ONB with lidocaine for diagnoses, were included in this study, all of whom were treated using dexamethasone and lidocaine. Pain intensity and ONB correlated complications and side effects were collected and analyzed at three different time points: before ONB, at 1 day after ONB, and at discharge.

**Results:**

Nineteen males and twenty-two females aged 49.6 ± 15.2 years were diagnosed with drug-resistant ON-APCH. The mean NRS was 8.0 ± 0.9 before ONB, which later significantly decreased to 2.1 ± 1.4 and 1.6 ± 0.6 at 1 day after ONB and on discharge, respectively. At 1 month after ONB, thirty patients (73%) obtained complete pain relief without medication. At 3 months after ONB, only two (5%) patients had to continue oral medications to maintain pain relief. No adverse effects or complications were observed immediately after, or within 3 months, of the nerve blockade.

**Conclusions:**

For drug-resistant ON-APCH, early occipital nerve blockade with dexamethasone and lidocaine is an effective and safe technique, which provides adequate pain relief and may prevent further development of persistent presentation of refractory ON.

## 1. Introduction

Occipital neuralgia (ON) is a neuropathic condition that mainly affects the occipital and suboccipital region. The pain is characterized by unilateral or bilateral throbbing, shooting or lancinating episodic aches, presenting along the greater, lesser, and third occipital nerve distribution or, sometimes, in an occipital-temporal-frontal distribution. Hypoesthesia or dysesthesia in the area of occipital nerve innervation, tenderness on palpation, and positive Tinel's sign are some of the major findings of ON. Moreover, severe neuralgic pain often presents with ocular pain, tinnitus, nausea, and dizziness. The accompanying symptoms of ON occur due to the nervous connections of occipital nerve with vestibulocochlear nerve, glossopharyngeal nerve, vagus nerve, and cervical sympathicus [1, 2]. Based on the current literature, 8.3% of all facial pain is reported to be ON, with an incidence of 3.2 per 100,000 people [3]. 90% of all ON is correlated with pathophysiological changes of the greater occipital nerve, and the other 10% is due to the damage of lesser occipital nerve. The third occipital nerve rarely results in ONs [4]. Greater occipital nerve and lesser occipital nerve receive sensory fibers from C2 and C2/C3 nerve roots, respectively. Typical causes of ON include nerve entrapment, trauma, inflammation, and whiplash [5].

Studies suggest that, following craniotomy, patients are likely to develop persistent ON, which is an uncommon subclassification of chronic postcraniotomy headache (PCH). According to the Headache Classification Committee of the International Headache Society (IHS), the International Classification of Headache Disorders, 3rd edition, PCH is defined as acute or chronic headache attributed to craniotomy [6]. Pericranial muscular adherence and aseptic meningitis are believed to be two major factors leading to PCH [7]. The characterization of PCH varies in many ways. Symptoms are described as incisional [8], throbbing [9], pressing [10], and sometimes neuropathic [11]. Levo et al. noticed that, after acoustic neuroma removal, some chronic PCHs originate from occipital nerve innervation and present as neuralgic headache in an occipital-temporal-frontal distribution [12]. In 2009, Schankin et al. reported that, 5 out of 30 patients with chronic PCH fit the diagnostic criteria of ON and presented the first report of persistent ON after acoustic neuroma surgery [13]. Thereafter, Ducic et al. reported that persistent drug-refractory ON following acoustic neuroma resection can be relieved by occipital nerve excision [14]. Ducic et al. described that 6 out of 7 patients experienced pain reduction and improvement of more than 80% on migraine index and quality of life [14]. This finding confirmed that ON following acoustic neuroma surgery was associated with a nerve entrapment or neuroma compression due to surgical incision [14]. Additionally, a recent study reported that nerve allograft was positive for drug-resistant persistent ON caused by lesser occipital nerve damage, during a retrosigmoid craniotomy [15]. Investigations suggest that persistent pain caused by intraoperative impairment of occipital nerves seems to be an uncommon but intractable problem.

Conservative treatments failed in most of the above cases. Most patients must undergo surgical management for persistent ON. It is assumed that early management of ON-like acute postcraniotomy headache (ON-APCH) after craniotomy might better relieve pain and help prevent the development of persistent ON. However, based on the current literature, little is known about ON-APCH and its management. In this study, medical data of 41 patients who developed drug-resistant acute PCH, similar in nature to occipital neuralgia, within 7 days after craniotomy, and received occipital nerve blockade (ONB) using dexamethasone and lidocaine, were retrospectively collected and analyzed. The therapeutic effectiveness and safety of ONB on ON-APCH was evaluated.

## 2. Patients and Methods

### 2.1. Population and Eligibility

Patients' medical documents and case records between January 2012 and December 2020 were reviewed. Patients who fulfilled the following criteria were included in this study: underwent elective craniotomy for intracranial disease, asked for a consultation with the institutional pain management center, diagnosed with ON-APCH following craniotomy within 1 week as described by the Headache Classification Committee of the IHS, the International Classification of Headache Disorders (version 2, 3-beta, and 3 based on the time of diagnosis) [6, 16, 17], presented with severe pain that could not be relieved by oral analgesics, and received therapeutic ONB by pain physicians. Patients with any of the following criteria were excluded from the study: confirmed with ON before craniotomy, underwent other invasive treatment for ON-APCH before ONB, and had incomplete clinical records.

### 2.2. Diagnosis and Treatment

For patients who underwent elective craniotomy, routine postoperative analgesia through oral analgesics, and patient-controlled intravenous analgesia (PCIA), containing opiates and antiemetics, were given as recommended, on the first postoperative day [18]. PCIA was removed before the end of the second postoperative day. When patients reported a drug-resistant postoperative pain or headache after craniotomy, after intracranial pathophysiological changes were ruled out, consultation with a pain physician was asked by the neurosurgeons. If symptoms and examinations suggested ON, diagnostic blockade of the greater and/or lesser occipital nerve was performed by the pain physician to confirm ON-APCH. Therapeutic ONB was performed for patients who responded positively to the diagnostic blockade. After nerve blockades, oral analgesics were continued or discontinued as per the evaluation of the pain physician.

### 2.3. Nerve Blockade Technique

Patients were asked to be seated with their head forward, positioning the ears in front of the body's vertical line. The injection point of greater occipital nerve was identified by palpation, and superior nuchal ridge was palpated to confirm occipital artery pulsation. Occipital nerve is located medial to the occipital artery, approximately 2 cm lateral and 2 cm inferior to occipital protuberance. After sterilization, a 5 ml syringe, with 25–27-gauge needle of approximately 1–1.5-inch, was orthogonally inserted to a depth of 3-4 mm until stopped by the periosteum. Once the needle was stopped, it was withdrawn 1 mm backward and then reoriented cephalad. A fanlike injection was performed after aspiration. For lesser occipital nerve blockade, the puncture site was localized approximately 3-4 cm lateral and 0.5–1 cm caudal to the former site. The optimal position was confirmed by palpation as well. Sterilization, aspiration, and injection procedure were identical to ONB. For diagnostic blockade, 1% lidocaine was administered at each puncture point. As to therapeutic blockade, the injection was formulated with 1% lidocaine and dexamethasone (4 mg), diluted with normal saline. The solution was injected into the affected nerves based on the evaluation of pain distribution and intensity [19, 20].

### 2.4. Data Collection and Evaluation

Data were collected by two research assistants by reviewing consultation records, case records, and institutional electronic medical record system. Preoperative baseline demographics were obtained from the electronic medical record system, which included gender, age, preoperative comorbidities, or headache disorders. Perioperative data were collected from the operation and anesthesia records, including preoperative regional management, side of surgical incision (right or left), reason for surgery, surgical approach, and duration of operation. Data on time of onset of ON, symptoms of pain presentation, pain intensity, analgesics requirement (required or not required), time-to-treat (defined as the time interval from the onset of pain to ONB), side effects, or complications attributed to ONB were collected from case records. Routine revision and follow-up were scheduled one day after consultation. During hospitalization, a pain specialist made regular follow-up visits, one day after consultation, and before discharge, in order to observe the effects of treatment. After discharge, routine follow-up through telephone interviews were scheduled at 1 and 3 month(s) after ONB. Pain intensity was determined using a Numerical Rating Scale (NRS) during hospitalization, in which 0 indicated no pain and 10 indicated the worst pain imaginable, and the Barrow Neurological Institute (BNI) pain inventory after discharge, which is shown in Table 1.

### 2.5. Statistical Analyses

All data were analyzed using SPSS version 25.0. Normality was examined using the Shapiro–Wilk test. Normally distributed continuous variables were presented with mean and standard deviation. Skewed data were presented with median (range). Categorical variables were recorded as frequency (percentage). NRS before and after ONB was compared using the one-way repeated-measures analysis of variance (ANOVA). To evaluate the influence of timing of ONB on therapeutic effectiveness, two subgroups were formed based on time-to-treat (≤7 or≥8 days) with two-way repeated-measures ANOVA. *P* < 0.05 was determined for statistical significance.

## 3. Results

We retrospectively reviewed institutional medical records from January 2012 to December 2020. A total of 51 patients fulfilled the inclusion criteria; four were confirmed to have pre-existing ON, and one had received acupuncture in the suboccipital region before consultation. Incomplete data due to loss of follow-up was recorded in five patients. Ultimately, 41 patients were included in the present study. Baseline demographics and perioperative data of eligible patients are shown in Table 2.

After craniotomy, ipsilateral ON-APCH was found in 38 of the 41 patients. 3 patients developed bilateral ON-APCH postoperatively. The onset of symptoms was 3.5 ± 1.3 days after craniotomy. 22 patients complained of throbbing and/or stabbing pain. The other 19 patients described the pain to be indistinguishable. The median time-to-treat was 6 (2–11) days. Unilateral greater and lesser occipital nerve blockade was performed in 13 and 17 patients, respectively. Unilateral blockade for both greater and lesser was performed in 8 patients. 3 patients received bilateral greater occipital nerve blockade.

**Figure 1:**
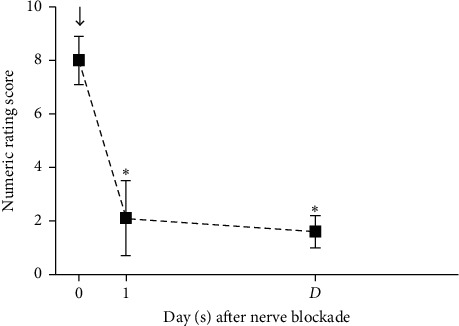
Numeric rating scores in 41 patients before and after occipital nerve blockade. Data at each time point were presented with mean and standard deviation. ↓: occipital nerve blockade. *∗*, *P* < 0.001 vs. baseline. D: discharge.

The difference in mean NRS at each time point was statistically different (df = 1.4, *F* = 444.3, *P* < 0.001). NRS was 8.0 ± 0.9 before ONB, 2.1 ± 1.4 at one day after ONB, and 1.6 ± 0.6 at discharge (Figure 1). There was a statistically significant difference in NRS at 1 day after ONB and at discharge, compared to NRS before ONB.

All patients received analgesics administration before ONB. 25 (61%) patients discontinued analgesics one day after ONB, while only 11 (27%) patients continued analgesics use after discharge.

**Figure 2:**
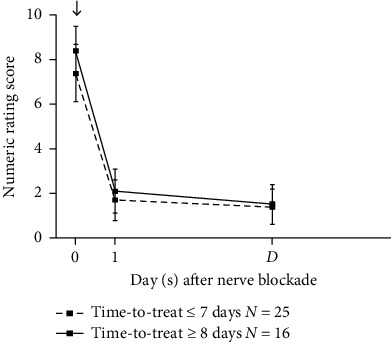
Numeric rating scores for the time-to-treat. Data at each time point were presented with mean and standard deviation. Time-to-treat: time interval from pain onset to occipital nerve blockade. ↓: occipital nerve blockade. Interaction concerning time-to-treat was not detected by the two-way repeated-measures ANOVA (df = 2, *F* = 1.984, *P*=0.152). D: discharge.

To determine the impact of ONB timing on therapeutic effectiveness, patients were divided into two groups based on time-to-treat (≤7 or≥8 days). Twenty-five patients received ONB within 7 days after ON onset, while the other sixteen underwent treatment beyond 8 days. NRS before ONB, at 1 day after ONB, and at discharge were compared between the two subgroups. Although longer time-to-treat was associated with a higher NRS at each time point, the difference between the groups were not statistically significant (Figure 2) (df = 2, *F* = 1.984, *P*=0.152). NRS fell analogously at each time point, regardless of the timing of ONB administration after craniotomy.

**Figure 3:**
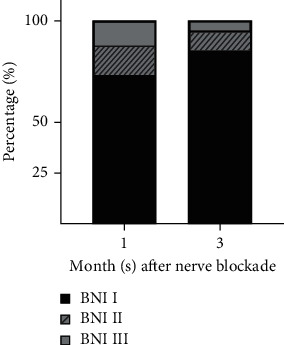
Pain intensity at 1 and 3 month(s) after occipital nerve blockade. BNI: Barrow Neurological Institution pain inventory.

The effectiveness of ONB during follow-up period is described in Figure 3. All 41 patients received stable pain relief with or without medication (BNI I-III) during the follow-up period. At 1 month after ONB, thirty (73%) patients experienced no pain without medication (BNI I), while five (12%) patients had to take oral analgesics (BNI III). Six (15%) patients experienced mild pain that did not require medication (BNI II). At 3 months after ONB, only two (5%) patients required medication to maintain pain relief (BNI III). Thirty-five (85%) patients experienced no pain without medication (BNI I), while four (10%) patients experienced mild pain that did not require analgesics (BNI II).

Neither immediate nor extended adverse effects or complications, including transient dizziness, intravenous injection, sudden unconsciousness, cardiovascular reactions, peri-injection alopecia, gastric ulcers, and other steroid-related adverse effects were observed after ONB throughout the study period.

## 4. Discussion

In this study, we report the therapeutic effectiveness and safety of occipital nerve blockade (ONB) with dexamethasone and lidocaine in 41 patients with drug-resistant ON-APCH, by reviewing consultation records and electronic medical data between January 2012 and December 2020. ON following craniotomy is deemed an uncommon subclassification of PCH. Ever since Schankin et al. first subclassified ON following craniotomy [7], persistent ON following craniotomy has occasionally been reported. However, little has been reported regarding acute occipital neuralgia-like postcraniotomy headache. To the best of our knowledge, this is the first study on ON-APCH and the therapeutic effectiveness and safety of ONB with dexamethasone and lidocaine in treating ON-APCH.

In line with the management of ON, treatment of ON-APCH includes conservative treatment and interventional procedures. Conservative treatments including physical therapies, rest, heat, massage, and nonsteroidal anti-inflammatory drugs (NSAIDs) remain the first-line therapy. For drug-resistant patients, interventional procedures are performed. In the current study, significant improvement was observed in all patients after therapeutic ONB procedure. Compared to the NRS before ONB, mean NRS decreased by 74% at 1 day after ONB and 93% at discharge. Approximately 40% of patients stopped oral analgesic medications at day 1 after ONB. The result is in line with a previous study on ONB treating drug-resistant ON [21]. Evident pain relief was maintained at discharge (1.6 ± 0.6 on NRS), and only 28% of patients had to continue oral analgesic for satisfactory pain relief. Oral analgesia was needed by 12% and 5% of patients at 1 month and 3 months after ONB, respectively. A majority of patients developed no pain or slight pain without medications during the 3-month follow-up period. No recurrent drug-resistant ON was observed. In a recent case review, symptomatic pain relief that was maintained for more than 3 months after ONB was reported in 74% of patients with persistent ON [22]. The effective rate was relatively higher in our findings. This is probably because patients' symptoms were managed within 6 (2–11) days of onset, in our study. Subgroup analyses revealed that promising therapeutic effect was observed in all patients after ONB regardless of time-to-treat. Though higher NRS before ONB was reported among patients with time-to-treat longer than 7 days, statistical difference was not observed between the two subgroups (≤7 or≥8 days) by two-way repeated-measures ANOVA. The effectiveness of ONB was predictably excellent, as all patients received ONB at an early stage of pain onset (within 11 days), though higher NRS was observed in patients with longer time-to-treat. Based on this trend, we speculate that ONB would be less effective in persistent ON. For persistent presentations of ON, pharmacological treatment including serotonin reuptake inhibitors, tricyclic antidepressants and anticonvulsants, and even surgical techniques like occipital nerve excision can be used. None of the 41 patients included in this study developed persistent ON throughout the follow-up period. Our results reveal that early diagnosis and management of ON-APCH provides reliable pain relief and may prevent further development of persistent presentation of refractory ON.

Although imaging guidance was not applied, ONB procedures were performed by skilled physicians, and injection sites were confirmed by anatomical location and physical examinations. Dexamethasone, as recommended and used in previous literatures [21, 23], was conservatively administered (not exceed 4 mg), to avoid excessive systemic absorption of corticosteroids. Throughout the course of the study, side effects or complications of dexamethasone and nerve blockade were not observed, suggesting that ONB is a safe technique for ON-APCH.

Although the etiology of ON-APCH remains unclear, recent reports suggest perioperative trauma to be the cause of ON [22]. The underlying trauma to the occipital region in retrosigmoidal or suboccipital craniotomy is believed to result in ON. Chronic entrapment of occipital nerves caused by peri-incisional neuroma formation led to nerve injury, which leads to persistent ON after craniotomy. In this study, all patients with ON-APCH responded positively to ONB with lidocaine and dexamethasone. When administered along with lidocaine for nerve blockade, the pharmacological mechanism of dexamethasone mainly involves elimination of local inflammation. This suggests that inflammation of surrounding tissue caused by incisional damage might be an underlying cause of ON-APCH. However, this cannot explain why 3 patients developed bilateral ON after unilateral craniotomy. Occipital nerve excitation due to prolonged occipital muscle traction as well as a fixed position during craniotomy was suspected to promote bilateral ON.

This study has some potential limitations. According to the IHS International Classification of Headache Disorders (3rd version) [17], headaches that are reported to have developed within 7 days after craniotomy should be diagnosed as acute postcraniotomy headache (APCH). Although, APCH mostly occurs at the incision site, or as tension type headache or migraine [17], postcraniotomy patients who developed neuralgic pain in the occipital or suboccipital area were included in this study. Based on clinical presentation and physical examination, we speculate that the patients developed ON after craniotomy. In addition, the outcomes of ONB support the diagnosis of ON. However, it is difficult to diagnose ON based on the current version of classification of headache disorders [17]; therefore, the postcraniotomy pain observed in the present study is classified as ON-APCH. The sample size of our study was limited due to the low prevalence of ON-APCH. Because of its retrospective nature, all patients included in this study were screened for drug-resistant ON-APCH. Thus, the sample size was further reduced. Therefore, larger, multicentric, prospective research is necessary to identify the clinical presentations, managements, and prophylaxis of this special type of APCH. Moreover, long-term effectiveness of ONB was not investigated due to the lack of a prospective design. Notably, the results of our study should be taken with caution as no control group was set.

## 5. Conclusion

For drug-resistant ON-APCH cases, early ONB with dexamethasone and lidocaine is an effective and safe choice, which provides reliable pain relief and prevents further development of persistent presentation of refractory ON.

## Figures and Tables

**Table 1:** Barrow Neurological Institute pain inventory.

BNI degrees	Explanations
BNI I	No pain without medications
BNI II	Mild pain not requiring medications
BNI III	No or controlled pain with medications
BNI IV	Improved pain cannot be relieved by medications
BNI V	Persistent pain cannot be relieved by medications

BNI: Barrow Neurological Institute pain inventory.

**Table 2:** Baseline demographics and perioperative data (*n* = 41).

Characteristics	Values
Age (years)	49.6 ± 15.2

Gender	
Female	22 (53.7%)
Male	19 (46.3%)
Preoperative anxiety and/or depression	4 (9.8%)

Preoperative headache	
Tension-type headache	3 (7.3%)
Migraine	2 (4.9%)

Preoperative regional management	
Scalp nerve blockade	17 (41.5%)
Scalp infiltration	19 (46.3%)
N/A	5 (12.2%)

Side of surgical incision	
Left	21 (51.2%)
Right	20 (48.8%)

Etiology for surgery	
Tumor resection	18 (43.9%)
Decompression	13 (29.3%)
Others	10 (22.0%)

Surgical approach	
Retrosigmoid	23 (56.1%)
Suboccipital	16 (39.0%)
Others	2 (4.9%)
Operation duration (hours)	6.3 ± 1.1

## Data Availability

The datasets used and/or analyzed during the current study are available from the corresponding author on reasonable request.

## Abbreviations

ON: Occipital neuralgia
TON: Third occipital nerve
PCH: Postcraniotomy headache
IHS: International Headache Society
ONB: Occipital nerve blockade
IRB: Institutional review board
PCIA: Patient-controlled intravenous analgesia
NRS: Numerical Rating Scale
BNI: Barrow Neurological Institute pain inventory
ANOVA: Analysis of variance.
